# Enabling exercise prescription for survivors of cancer

**DOI:** 10.1038/s41598-021-89021-w

**Published:** 2021-05-05

**Authors:** Katherine R. White, Jana Lu, Zara Ibrahim, Priscilla A. Furth

**Affiliations:** 1 Georgetown University School of Medicine, 3900 Reservoir Rd. NW, Washington, DC 20007 USA; 2 Departments of Oncology and Medicine and Lombardi Comprehensive Cancer Center, Georgetown University Medical Center, Georgetown University, 3970 Reservoir Rd. NW, Research Bldg. Room E521, Washington, DC 20057 USA; 3 3970 Reservoir Rd NW, Research Building, Room 520A, Washington, DC 20057 USA

**Keywords:** Health care, Oncology, Risk factors

## Abstract

Although exercise is widely recommended for survivors of cancer, readily implementable approaches for evaluating exercise tolerance enabling exercise prescriptions at appropriate levels of cardiovascular exertion are not always available. We evaluated the utility of modified Harvard Step tests within the context of a standard physical examination for fitness evaluation and exercise prescription for survivors of cancer across a range of age, BMI and exercise history. While 52% of presenting individuals with a past cancer diagnosis were able to complete a 3-min test at pace with a reduced 9-in. step, adoption of self-determined pacing, test duration and completion on a flat surface enabled relative fitness rating and appropriate exercise prescription for the remaining survivors. Younger age and more vigorous exercise histories correlated with completion of the standard 3-min test at pace, but all 9-in. formats led to exercise prescriptions more vigorous than current activity. The physical examination setting expedited inclusion of core and specific muscle group strength testing. The approach is adaptable to a range of health care settings, providers, and patients, providing a shared opportunity for providers and patients to evaluate exercise tolerance. It can be used to further expand incorporation of exercise testing and prescription into routine care.

## Introduction

Survivors of cancer are a rapidly increasing population globally due to improved screening, diagnoses, and treatments. In the United States alone, there are more than 15.5 million survivors of cancer; this number is expected to double in a few decades^1^. For years, clinicians advised cancer patients to refrain from physical activity; however, in the late 1990s, research outlined the benefits of physical activity, including improving survivorship, physical well-being, and quality of life^1–3^. The American Cancer Society (ACS) and the American College of Sports Medicine (ACSM) recommend survivors of cancer engage in at least 150 min of moderate or 75 min of vigorous aerobic activity per week, with biweekly resistance training and daily muscle stretching^1,3^. At a minimum, current guidelines urge survivors of cancer to avoid inactivity. In 2018, the American Heart Association (AHA) emphasized the importance of integrating physical activity assessment in routine clinical practices and workflow^4^. Recent studies have found that survivors of cancer who exercise have lower relative risks of cancer mortality, improved quality of life, and reduced fatigue^5–8^. Despite these recommendations, up to 80% of survivors of cancer may not meet ACS and ACSM physical activity recommendations and both survivors and providers are not always aware of all the recommended exercises, which include aerobic, balance, stretching, and muscle and bone strengthening^2,3,9^.

Survivors of cancer face unique cardiovascular fitness challenges. Chemotherapy and radiation therapy may cause cardiotoxicity, potentially impairing exercise ability^10^. Patients with hormone receptor-positive operable breast cancer treated with chemo-endocrine therapy have lower peak exercise stroke volume, cardiac output, cardiac power output and reserve, and VO2_peak_ than their healthy counterparts^11^. Exercise interventions have been shown to increase fitness, physical function, and quality of life in survivors of prostate cancer treated with hormone therapy^12,13^. Survivors who are overweight or obese experience increased cancer risk and decreased overall survival^14^. Hypertension also increases risk of cancer mortality^15^. Although cancer patients with distant metastasis are not typically counseled on exercise, studies have found that exercise in advanced cancer can improve fitness, function, fatigue, and overall quality of life^16,17^.

It is important for survivors of cancer to consult a physician before engaging in physical fitness programs in order to clear the patient for appropriate exercise^1,3,18,19^. While exercise is possible to initiate at all ages, careful attention must be given for older individuals who may be at a greater risk of falls, injuries, sudden cardiac death or myocardial infarction^3,20^. If prescribed exercise is too intense, patients are more likely to drop out^21^. There are limited numbers of peer-reviewed studies addressing how primary care providers may provide exercise prescriptions, especially for survivors of cancer. This task is often left to the oncologist. Formal training in physical fitness assessment is frequently lacking in medical school curriculum and may be limited to specific medical specialties such as physical medicine and rehabilitation^22–24^. Approaches for fitness assessment in the office setting include self-report or direct fitness testing^25^. Comprehensive fitness assessment should assign intensity to past and current exercise history, cardiovascular fitness, muscle strength, balance, and flexibility^26^. Self-reported physical activity can be coded with respective Metabolic Equivalency of Task (MET), a reflection of required oxygen uptake^27^.

The Harvard Step Test is one tool for fitness assessment. Since its initial development for evaluation of military recruits in World War II, the basic technique has been shown to be modifiable and applicable to different settings^28^. The Young Men's Christian Association (YMCA) provides age and gender-adjusted standards for fitness rating using 1 min pulse recovery standard for a 12-in.-3-min-96 steps/min format^29^. Correlates of fitness in adults include positive associations with male sex, education, socioeconomic status, and leisure-time physical activity and inverse relationships with increased age, body mass index (BMI), resting heart rate and higher blood pressure^30,31^.

Step tests require more exertion than a walk test making them a better choice for patients with higher baseline fitness levels and can be modified by step height and duration for use in special populations^32,33^. Our retrospective chart review evaluated the impact of utilizing a 3-min 9-in. step test with survivor-driven modifications for fitness evaluation in survivors of cancer with reference to how results correlated with past and current exercise histories. A group unable to complete a step were afforded a survivor-modifiable pace and time step-in-place on the floor (flat test). The step test was performed as part of a comprehensive physical examination that included evaluation of neurological and muscular functioning. This involved testing the utility of submaximal (max) muscle strength performance using a 20-max-crunch test for evaluation of abdominal muscle endurance, 20-max-modified-push-ups from the knees for evaluation of overall muscle strength and endurance, a 20-s-max plank (prone bridge) for core strength, and a 5-max-squat test for evaluation lower extremity muscle weakness and tightness^34–36^.

## Results

### Correlates of fitness included higher education, socioeconomic status and cardiorespiratory parameters

In adults, higher fitness levels have been correlated with higher education and socioeconomic status, absence of hypertension and elevated resting pulse^30,31^. The majority of survivors in this study were actively employed at the time of presentation (76%), largely within Education, Research and Development (21%) and Service economic sectors (60%) (Table 1). Individuals uniformly held established places of residence with living situations divided across living with a life partner (34%), living with family including children (32%), and living alone (29%). For some individuals information was not available in the chart (10% for employment status, 19% for employment, 4% for living situation). Initial resting office pulse and systolic blood pressures means were within the normal range with scattered outliers and there were no significant differences between step test groups or from the group performing a flat test or groups for whom a step test was either contraindicated or deferred (Table 2).

**Table 1:** Demographic characteristics.

Clinic total	171
Cancer survivor total, no. (%)	169 (100)
**Employment status, no. (%)**	
Currently working	128 (76)
Not currently working	23 (14)
Information unavailable	18 (10)
**Economic sector, no. (%)**	
Service	102 (60)
Business/finance/consultant	20
Art/design/media/journalism	16
Administration/HR/manager/director	20
Healthcare	19
Public/government service	15
Law	12
Education, research and development	35 (21)
Education/academia	21
Science/technology/engineering	14
Information unavailable	33 (19)
Residence, no. (%)	
Established place of residence	169 (100)
**Living situation, no. (%)**	
Living with life partner	57 (34)
Living with family including children	55 (32)
Living alone	49 (29)
Living with roommate	1 (1)
Information unavailable	7 (4)

**Table 2:** Fitness correlates with exercise history.

Total cohort		Test completed				Contraindicated	Deferred
		At pace	Slower self-paced	Shorter self-paced	Flat self-paced		
Cancer survivors Number (%)	169 (100)	88 (52)	24 (14)	21 (12)	9 (5)	20 (12)	7 (4)
Initial office pulse, beats/min mean ± SEM (range)	83 ± 1.0 (56–117)	83 ± 1.4 (56–117)	82 ± 1.9 (64–97)	86 ± 3.1 (58–114)	85 ± 5.5 (65–114)	83 ± 3.0 (61–105)	76 ± 3.6 (66–90)
Initial office systolic blood pressure, mmHg mean ± SEM (range)	126 ± 1.2 (94–188)	124 ± 1.5 (102–171)	131 ± 4.5 (101–188)	130 ± 3.1 (98–153)	124 ± 6.9 (98–164)	125 ± 3.7 (104–160)	124 ± 5.4 (94–138)
BMI, kg/m^2^ mean ± SEM (range)	28 ± 0.5 (17–44)	26 ± 0.6 (19–44)	28 ± 1.1 (19–42)	30 ± 1.3 (17–39)	31 ± 3.3 (19–43)	30 ± 1.5 (18–44)	23 ± 1.3 (18–27)
**Past exercise history**^**a,b**^** number (%)**							
Sedentary	28 (16)	14 (16)	5 (21)	4 (19)	1 (11)	3 (15)	1 (14)
Light	40 (24)	19 (22)	6 (25)	9 (43)	4 (44)	2 (10)	0 (0)
Moderate	44 (26)	15 (17)	6 (25)	6 (29)	4 (44)	12 (60)	1 (14)
Vigorous	57 (34)	40 (45)	7 (29)	2 (10)	0 (0)	3 (15)	5 (72)
**Current exercise history**^**c**^** number (%)**							
Sedentary	57 (34)	20 (23)	12 (50)	11 (52)	2 (22)	12 (60)	0 (0)
Light	64 (38)	37 (42)	9 (38)	6 (29)	7 (78)	3 (15)	2 (29)
Moderate	31 (18)	19 (22)	3 (13)	4 (19)	0 (0)	5 (25)	0 (0)
Vigorous	17 (10)	12 (14)	0 (0)	0 (0)	0 (0)	0 (0)	5 (71)

^a^Past exercise Hx: at pace vs. shorter self-paced *p* = 0.0191, chi square, two-tailed. At pace vs. flat self-paced *p* = 0.0078, Fisher's exact, two-tailed. At pace vs. contraindicated *p* = 0.0007, chi square, two-tailed.

^b^Past exercise vs. current exercise Hx: total cohort *p* = 0.0000, at pace *p* = 0.0000, chi square, two-tailed. Slower self-paced *p* = 0.0081, contraindicated *p* = 0.0042, Fisher's exact, two-tailed.

^c^Current exercise Hx: at pace vs. slower self-paced *p* = 0.0304, at pace vs. shorter self-paced *p* = 0.0355, at pace vs. flat self-paced *p* = 0.0014, at pace vs. contraindicated *p* = 0.0036, at pace vs. deferred *p* = 0.0043, Fisher's exact, two-tailed.

### Challenges to fitness included higher BMI, increased sedentary activity, advancing age, female gender and multiple cancer treatment modalities

In adults lower fitness levels have been correlated with higher BMI, increased sedentary activity, higher age, and female gender^30^. Cancer diagnosis has been reported to be associated with increased sedentary behavior^37^. Mean BMI for study participants was in the overweight range (28 kg/m^2^ ± 0.46, mean ± SEM, range 17–44 kg/m^2^) without significant variation between groups (Table 2). Thirty-four percent described their current exercise activity as sedentary, a significant difference from only 16% who described their past exercise history as sedentary (p < 0.00001, Chi Square, two-tailed) (Table 2). Only 10% described their current exercise as vigorous whereas 34% characterized past exercise as vigorous (p < 0.00001, Chi Square, two-tailed) (Table 2). Survivors who described more vigorous past and/or current exercise histories were more likely to complete the 3-min 9-in. step test at pace as compared to the other less vigorous step test formats (*p* < 0.02, Chi Square, two-tailed, *p* < 0.04 Fisher’s Exact, two-tailed) (Table 2). Mean age of the studied population was 53 ± 1 years (range 27–79 years) (Table 3).

**Table 3:** Age, gender and cancer characteristics.

Total cohort		Test completed				Contra-indicated	Deferred
		At pace	Slower self-paced	Shorter self-paced	Flat self-paced		
Cancer survivors Number (%)	169 (100)	88 (52)	24 (14)	21 (12)	9 (5)	20 (12)	7 (4)
Age at clinic presentation^a^, years mean ± SEM (range)	53 ± 1 (27–79)	49 ± 1 (27–66)	56 ± 2 (38–74)	60 ± 2 (39–77)	65 ± 3 (54–76)	58 ± 3 (35–79)	43 ± 2 (37–51)
Age at cancer diagnosis^b^, years mean ± SEM (range)	51 ± 1 (26–78)	47 ± 1 (26–64)	54 ± 2 (29–74)	59 ± 2 (38–76)	63 ± 3 (50–76)	56 ± 3 (35–78)	40 ± 2 (34–46)
Years post cancer diagnosis, mean ± SEM (range)	1.8 ± 0.2 (0–26)	1.7 ± 0.4 (0–26)	2.1 ± 0.7 (0–13)	2.1 ± 1.0 (0–22)	1.9 ± 0.8 (0–8)	1.4 ± 0.4 (0–6)	2.3 ± 0.9 (0–6)
Female	159 (94)	85 (97)	22 (92)	21 (100)	9 (100)	15 (75)	7 (100)
Male	10 (6)	3 (3)	2 (8)	0 (0)	0 (0)	5 (25)	0 (0)
**Active chemotherapy number (%)**							
Yes	47 (28)	23 (26)	5 (21)	7 (33)	2 (22)	10 (50)	0 (0)
No	122 (72)	65 (74)	19 (79)	14 (67)	7 (78)	10 (50)	7 (100)
**Active anti-hormonal therapy number (%)**							
Yes	82 (48)	45 (51)	11 (46)	9 (43)	4 (44)	8 (40)	5 (71)
No	87 (52)	43 (49)	13 (54)	12 (57)	5 (56)	12 (60)	2 (29)
**Radiation therapy number (%)**							
Completed	69 (41)	27 (31)	14 (58)	12 (57)	5 (56)	8 (40)	3 (43)
None	97 (57)	58 (66)	10 (42)	9 (43)	4 (44)	12 (60)	4 (57)
Active	3 (2)	3 (3)	0 (0)	0 (0)	0 (0)	0 (0)	0 (0)
**Surgical procedure number (%)**							
Completed	158 (93)	83 (94)	22 (92)	21 (100)	7 (78)	18 (90)	7 (100)
None	8 (5)	3 (3)	1 (4)	0 (0)	2 (22)	2 (10)	0 (0)
Pending	3 (2)	2 (2)	1 (4)	0 (0)	0 (0)	0 (0)	0 (0)
**Cancer diagnosis number (%)**							
Breast	149 (87)	83 (94)	20 (83)	19 (90)	7 (78)	13 (65)	7 (100)
Gastrointestinal	6 (4)	2 (2)	1 (4)	1 (5)	1 (11)	1 (5)	0 (0)
Head and neck	6 (4)	2 (2)	2 (8)	0 (0)	0 (0)	2 (10)	0 (0)
Genitourinary	4 (2)	0 (0)	1 (4)	0 (0)	0 (0)	3 (15)	0 (0)
Hematopoietic	3 (2)	1 (1)	0 (0)	0 (0)	1 (11)	1 (5)	0 (0)
Lung	1 (1)	0 (0)	0 (0)	1 (5)	0 (0)	0 (0)	0 (0)
**Distant metastases**^**c**^** number (%)**							
Yes	16 (9)	7 (8)	1 (4)	2 (10)	0 (0)	6 (30)	0 (0)
No	153 (91)	81 (92)	23 (96)	19 (90)	9 (100)	14 (70)	7 (100)

^a^Age at clinic presentation: at pace vs. slower self-paced *p* = 0.0155, at pace vs. shorter self-paced *p* < 0.0001, at pace vs. flat self-paced *p* < 0.0001, at pace vs. contraindicated *p* = 0.0015, one-way ANOVA, Tukey's multiple comparisons test.

^b^Age at diagnosis: at pace vs. slower self-paced *p* = 0.0371, at pace vs. shorter self-paced *p* < 0.0001, at pace vs. flat self-paced *p* < 0.0001, at pace vs. contraindicated *p* = 0.0015, one-way ANOVA, Tukey's multiple comparisons test.

^c^Distant metastases: at pace vs. contraindicated *p* = 0.0142, Fisher's exact, two-tailed. SEM: standard error of the mean.

Younger age at clinic presentation was significantly correlated with the ability to complete a 3-min 9-in. step test at pace as compared to other test groups and for whom cardiovascular fitness testing was contraindicated (*p* < 0.05, One-way ANOVA, Tukey's multiple comparisons test, Table 3). The deferred group, survivors who were not step tested due to provision of documented high fitness activity, were also significantly younger than these other groups (*p* < 0.02, One-way ANOVA, Tukey's multiple comparisons test). Ninety-four percent of the survivors tested identified as female.

Chemotherapy, radiation and hormone therapy all have been reported to have a negative impact on cardiorespiratory fitness^10,11^. In the population studied 28% were on active chemotherapy, 48% on active anti-hormonal therapy and 2% under active radiation therapy (Table 3). Forty-one percent had completed radiation therapy and 93% completed at least one surgical procedure. The majority of survivors of cancer presented within two years of diagnosis (range: 0–26 years post cancer diagnosis). Similar to age at presentation, lower age at cancer diagnosis was positively correlated with the ability to complete a 3-min. 9-in. step test at pace (*p* < 0.05, One-way ANOVA, Tukey's multiple comparisons test). Breast cancer was the most common cancer diagnosis reported (87%). There is a strong emphasis on fitness preservation within the breast cancer patient, research, and provider communities^6,7,11^. The remaining cancer diagnoses were divided between Gastrointestinal (GI), Head and Neck, Genitourinary (GU), Hematopoietic and Lung cancers.

### Both cancer-related and non-cancer-related co-morbidities impacted fitness testing

Survivors of cancer are recommended to seek health provider clearance before engaging in exercise due to the possibility of cancer-related co-morbidities that could impact the safety of initiating an exercise program^1,3,18–20^. Here 44% of the survivors presenting to clinic were diagnosed with co-morbidities that required either modification of the standard testing protocol (32%) or step testing under any conditions was contraindicated (12%) (Table 4). Significantly 78% of the co-morbidities identified were not directly cancer-related. The leading reason for step test modification/contraindication was deconditioning (34%), which could be indirectly related to cancer due to a change in exercise routine proximal to the cancer diagnosis. To address that question survivors’ exercise past and current histories were queried. Sixty percent of deconditioned survivors provided lifetime histories of sedentary or light exercise behavior whereas for 40% the cancer diagnosis may have contributed to deconditioning as these individuals experienced a drop in exercise intensity level. The second most common reason was chronic knee pain (26%). In recent decades, prevalence of knee pain and osteoarthritis has increased significantly in the US, suggesting the high proportion found here is simply reflective of the relatively high incidence in the general population^38,39^. The presence of distant metastases, found in 9% of presenting survivors, also significantly impacted fitness testing as it was over-represented in survivors for whom a step test was medically contraindicated as compared to those that completed a 3-min 9-in. step test at pace (*p* = 0.0142, Fisher's Exact, two-tailed) (Table 3).

**Table 4:** Co-morbidities requiring step test modification or contraindication.

Modified/contraindicated tests		Modified tests			Contraindicated
		Slower self-paced	Shorter self-paced	Flat self-paced	
Cancer survivors number	74/169	24	21	9	20
**Cancer-related number from population requiring modification/contraindicated**	16/74	5	7	0	4
Chemotherapy-related fatigue/anemia	6	4	1	0	1
Post-chemotherapy neuropathy	3	1	2	0	0
Pulmonary metastases	2	0	2	0	0
Post-surgical pain/discomfort	2	0	1	0	1
Lower-extremity lymphedema	1	0	1	0	0
Onycholysis	1	0	0	0	1
Wound dehiscence	1	0	0	0	1
**Non-cancer related**	58/74	19	14	9	16
Deconditioned/sedentary behavior history	25	15	7	0	3
Chronic knee pain	19	3	3	7	6
Impaired balance	4	0	1	2	1
Chronic back pain	3	0	0	0	3
Cardiac stress test indicated	3	0	1	0	2
Chronic hip pain	2	1	0	0	1
Chronic pulmonary disease	1	0	1	0	0
Leg prosthesis	1	0	1	0	0

### Comparison of relative fitness rating stratification from the different test formats

**Figure 1:**
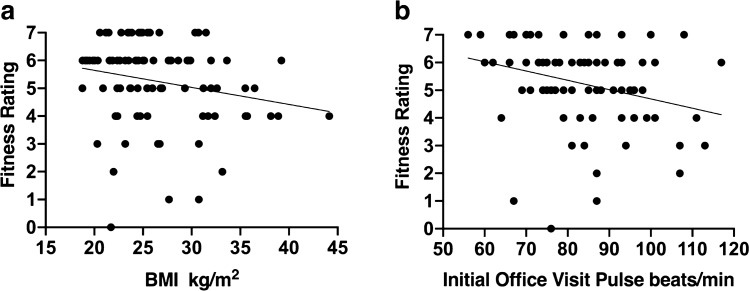
Fitness ratings negatively correlated with BMI and initial office pulse. (**A**) Regression scatter plot between fitness rating and BMI (kg/m^2^). Fitness rating decreased 0.061 per kg/m^2^ (F(1,86) = 4.149, *p* < 0.05), R^2^ 0.046. (**B**) Regression scatter plot between fitness rating and initial office pulse (beats/min). Fitness rating decreased 0.0336 for each beat/min (F(1,87) = 7.677, *p* < 0.05), R^2^ 0.081. Data from 3-min 9-in. At Pace group shown.

The YMCA developed relative fitness ratings for a 3-min 12-in. step test using the total 1-min post-exercise pulse recovery^29^. Here we tested if the age and gender adjusted ratings developed by the YMCA could be used to stratify relative fitness for the modified formats applied here (Table 5). Stratification was most robust for the group completing the 3-min 9-in. step test At Pace as it encompassed the full range from Very Poor to Excellent. The range of stratification contracted when survivors were allowed to self-modify pace and duration for a less strenuous test, but still provided relative scales ranging from Below Average to Excellent for both the Slower and Shorter Self-Paced groups and Average to Excellent for the Flat Self-Paced groups. This could effectively be used to generate appropriate personalized exercise prescriptions as exercise tolerance was directly observed, even when Excellent or Very Good for the formal Fitness Rating was an over-estimation. Chemotherapy and anti-hormonal therapy can impact fitness^11^. In the 3-min 9-in. At Pace group, fitness ratings were positively correlated with active hormone therapy (*p* = 0.0347, Fisher's Exact, two-sided) and negatively correlated with active chemotherapy (*p* = 0.0018, Fisher's Exact, two-sided) with significant regression equations for BMI and initial office pulse (Table 5, Fig. 1). Neither radiation therapy nor presence of distant metastases showed any significant associations with fitness ratings in any group.

**Table 5:** Fitness ratings.

Fitness ratings	Very poor-poor	Below average	Average	Above average	Good	Excellent
**Completed at pace (96 ± 0 steps/min (mean ± SEM, range 96), 9 in.) number (%)**						
Total number in group n = 87^a^	4 (5)	5 (6)	13 (15)	18 (21)	30 (34)	17 (19)
Active chemotherapy^b^						
Yes	1 (4)	3 (13)	9 (39)	3 (13)	4 (17)	3 (13)
No	3 (5)	2 (3)	4 (6)	15 (23)	26 (41)	14 (22)
Active anti-hormonal therapy^c^						
Yes	2 (4)	2 (4)	2 (4)	12 (27)	15 (33)	12 (27)
No	2 (5)	3 (7)	11 (26)	6 (14)	15 (36)	5 (12)
Radiation therapy						
Completed	2 (8)	0 (0)	5 (19)	6 (23)	8 (31)	5 (19)
None	2 (3)	5 (9)	8 (14)	10 (17)	21 (36)	12 (21)
Active	0 (0)	0 (0)	0 (0)	2 (67)	1 (33)	0 (0)
Presence of distant metastases						
Yes	0 (0)	2 (29)	1 (14)	2 (29)	1 (14)	1 (14)
No	4 (5)	3 (4)	12 (15)	16 (20)	29 (36)	16 (20)
**Completed slower self-paced (85 ± 1.1 steps/min (mean ± SEM, range 70–90), 9 in.) number (%)**						
Total number in group n = 24	0 (0)	1 (4)	1 (4)	7 (29)	9 (38)	6 (25)
Active chemotherapy						
Yes	0 (0)	0 (0)	1 (20)	1 (20)	3 (60)	0 (0)
No	0 (0)	1 (5)	0 (0)	5 (26)	7 (37)	6 (32)
Active anti-hormonal therapy^d^						
Yes	0 (0)	1 (10)	0 (0)	5 (45)	5 (45)	0 (0)
No	0 (0)	0 (0)	1 (8)	2 (15)	4 (31)	6 (46)
Radiation therapy						
Completed	0 (0)	1 (7)	0 (0)	5 (36)	4 (29)	4 (29)
None	0 (0)	0 (0)	1 (10)	2 (20)	5 (50)	2 (20)
Active	0 (0)	0 (0)	0 (0)	0 (0)	0 (0)	0 (0)
Presence of distant metastases						
Yes	0 (0)	0 (0)	0 (0)	0 (0)	1 (100)	0 (0)
No	0 (0)	1 (4)	1 (4)	7 (30)	8 (35)	6 (16)
**Completed shorter self-paced (91 ± 1.5 steps/min (mean ± SEM, range 80–96), 9 in.) number (%)**						
Total number in group n = 20^e^	0	3 (15)	2 (10)	5 (25)	6 (30)	4 (20)
Active chemotherapy^f^						
Yes	0 (0)	0 (0)	0 (0)	0 (0)	5 (71)	2 (29)
No	0 (0)	3 (23)	2 (15)	5 (38)	1 (8)	2 (15)
Active anti-hormonal therapy						
Yes	0 (0)	1 (13)	2 (25)	2 (25)	2 (25)	1 (13)
No	0 (0)	2 (17)	0 (0)	3 (25)	4 (33)	3 (25)
Radiation therapy						
Completed	0 (0)	2 (18)	2 (18)	2 (18)	3 (27)	2 (18)
None	0 (0)	1 (11)	0 (0)	3 (33)	3 (33)	2 (22)
Active	0 (0)	0 (0)	0 (0)	0 (0)	0 (0)	0 (0)
Presence of distant metastases						
Yes	0 (0)	0 (0)	1 (50)	0 (0)	1 (50)	0 (0)
No	0 (0)	3 (17)	1 (6)	5 (28)	5 (28)	4 (22)

^a^N = 1 excluded from analyses for exercise-induced arrhythmia.

^b^*p* = 0.0018, Fisher's exact, two-tailed.

^c^*p* = 0.0347, Fisher's exact, two-tailed.

^d^*p* = 0.0238, Fisher's exact, two-tailed.

^e^N = 1 excluded from analyses as pulse recovery not determined.

^f^*p* = 0.0103, Fisher's exact, two-tailed.

### Muscle group testing revealed strengths and weaknesses distinct from cardiovascular fitness

Although cardiorespiratory fitness is the factor most highly correlated with positive outcomes for survivors of cancer, core and extremity strength training are also recommended^1–8^. To answer this need, survivors of cancer were offered specific submaximal muscle group testing in addition to the more routine muscle strength evaluation completed as part of their comprehensive physical examination^34^. Step test performance was not necessarily predictive of strength testing (Table 6). For example, 56% of the survivors of cancer presenting to clinic attempted abdominal and hip-flexor strength testing with a crunch test, a percentage that was not significantly different across the different step test groups, and across all test groups the majority were able to complete the full 20 crunches in good form. Testing of three other muscle groups was explored in a smaller proportion of survivors. Relatively few (21%) of survivors were offered modified push-ups from the knees, in part due to the high prevalence of survivors of breast cancer in the clinic with limits on upper extremity weight bearing. However, even when attempted, the majority were able to complete less than ten, without significant difference across groups, and this test was dropped from the exam as a routine. A plank or prone bridge test, which assesses transversus abdominis, rectus abdominis, internal oblique and external oblique muscles was instituted as an alternative, completed by 10% of survivors^35^. The mean number of seconds held was 12 ± 2 s (mean ± SEM) with no significant variations between step test groups. Finally, due to the high prevalence of lower extremity co-morbidities (Table 4), use of a squat test was explored to assess quadriceps, hamstring and gluteal muscle strength and symmetry completed by 17% of the survivors^36^. Across all step test groups at least 50% had an abnormal test. Weaknesses that were revealed through specific muscle group testing were addressable through targeted exercises included in their exercise prescription and/or referral to physical therapy.

**Table 6:** Strength test performance.

Sub-maximal muscle testing		Test completed				Contraindicated	Deferred
		At pace	Slower self-paced	Shorter self-paced	Flat self-paced		
**Crunch test number (%)**							
Tested	94 (56)	55 (64)	14 (58)	15 (71)	5 (56)	3 (15)	2 (29)
Number performed							
20 (maximal)	82 (87)	50 (90)	12 (83)	12 (82)	3 (60)	3 (100)	2 (100)
15–19	3 (3)	1 (2)	0 (0)	1 (6)	1 (20)	0 (0)	0 (0)
10–14	3 (3)	1 (2)	2 (17)	0 (0)	0 (0)	0 (0)	0 (0)
5–9	4 (5)	3 (6)	0 (0)	1 (6)	0 (0)	0 (0)	0 (0)
0–4	2 (2)	0 (0)	0 (0)	1 (6)	1 (20)	0 (0)	0 (0)
**Push-up on knees number (%)**							
Tested	36 (21)	20 (23)	8 (33)	5 (24)	1 (11)	0 (0)	2 (25)
Number performed							
20 (maximal)	11 (30)	6 (30)	3 (37)	0 (0)	1 (100)	0 (0)	1 (50)
15–19	1 (3)	1 (5)	0 (0)	0 (0)	0 (0)	0 (0)	0 (0)
10–14	2 (6)	1 (5)	0 (0)	1 (20)	0 (0)	0 (0)	0 (0)
5–9	8 (22)	6 (30)	2 (26)	0 (0)	0 (0)	0 (0)	0 (0)
1–4	14 (39)	6 (30)	3 (37)	4 (80)	0 (0)	0 (0)	1 (50)
**Plank number (%)**							
Tested	17 (10)	8 (9)	2 (8)	5 (24)	1 (11)	1 (5)	0 (0)
Seconds held mean ± SEM (range)	12 ± 2 (5–30)	13 ± 3 (5–30)	10 ± 0 (10)	11 ± 2 (5–20)	15 ± 0 (15)	15 ± 0 (15)	
**Squat test number (%)**							
Tested	29 (17)	18 (20)	3 (12)	4 (19)	2 (22)	2 (11)	0 (0)
Normal result	10 (44)	7 (39)	1 (33)	0 (0)	1 (50)	1 (50)	
Abnormal result	19 (66)	11 (61)	2 (67)	4 (100)	1 (50)	1 (50)	

SEM: standard error of the mean.

### Exercise prescriptions received were more vigorous than current activity

Survivors of cancer, even those with disseminated disease, afforded the benefit of exercise programming show improvements in quality of life and physical function^5–8,12,13,15,16^. Here 91% of survivors were able to receive an exercise prescription, the majority receiving a prescription for exercise more vigorous than their current activity (p = 0.0000, Chi Square, two-tailed; p < 0.0004, Fisher’s Exact, two-tailed), Table 7). Health provider clearance is recommended prior to initiating a change in exercise for survivors clearance^1,3,18–20^. Eight survivors required cardiac stress test evaluation and two additional healing. There was a correlation between exercise prescription intensity and step test category completed. The 3-min 9-in. At Pace group receiving significantly more vigorous exercise prescriptions than other step test groups (*p* < 0.05, Fisher’s exact, two-tailed). Exercise prescriptions included both aerobic and resistance training. Aerobic training was tailored to survivor interest, experience and ability to promote engagement. Guidance on numbers of minutes and days per week to begin with were provided in concert with discussion on how the exercise program could be expanded as aerobic fitness improved. The resistance training portion of the exercise prescription was built upon needs identified through physical exam and specific muscle group testing and also included specific recommendations on numbers of repetitions per session, numbers of sessions per week, and how the training program could build as strength improved.

**Table 7:** Exercise prescription.

		Total cohort	Test completed						Contraindicated	Deferred
			At pace		Slower self-paced		Shorter self-paced	Flat self-paced		
Cancer survivors (total n per group)		169	88		24		21	9	20	7
		Number provided exercise prescription number (%)								
**Exercise prescription intensity**^**a,b**^ **Number (% column total)**		154 (91)	84 (95)		21 (88)		19 (90)	9 (100)	15 (75)	6 (86)
Sedentary		3	0		0		0	0	3^c^	0
Light		96	41		19		15	9	12	0
Moderate		41	33		2		4	0	0	2
Vigorous		14	10		0		0	0	0	4
		Number not provided exercise prescription number (%)								
**No exercise prescription** **Number (% column total)**		15 (9)	4 (5)		3 (12)		2 (10)	0 (0)	5 (25)	1 (14)
Consultation required before exercise Rx^d^		12	4		3		2	0	3	0
Healing required before exercise Rx^e^		2	0		0		0	0	2	0
Declined exercise Rx^f^		1	0		0		0	0	0	1

SEM: standard error of the mean.

^a^Exercise Rx: at pace vs. slower self-paced *p* = 0.0023, at pace vs. shorter self-paced *p* = 0.0483, at pace vs. flat self-paced *p* = 0.0190, at pace vs. contraindicated *p* = 0.0000, At pace vs. deferred *p* = 0.0012, Fisher's exact, two-tailed.

^b^Current exercise Hx (Table 2) vs. exercise Rx: total cohort *p* = 0.0000, at pace *p* = 0.0000, chi square, two-tailed. slower self-paced *p* = 0.0001, shorter self-paced *p* = 0.0002, contraindicated *p* = 0.0003, Fisher's exact, two-tailed.

^c^N = 1 balance exercises. N = 1 range of motion exercises. N = 1 activities of daily living only.

^d^N = 8 cardiac stress test, N = 1 each: exercise-induced cardiac arrythmia, MRI shoulder, Baker's cyst, left upper quadrant pain.

^e^N = 1 each: onycholysis, surgical incision.

^f^N = 1 came to clinic for tamoxifen consultation.

## Discussion

Modifiable step tests, administered in the context of a comprehensive physical examination that included exercise history and targeted testing of specific muscle groups, were successful in generating exercise prescriptions appropriate to fitness levels for survivors of cancer. History and physical provided preliminary evaluation of fitness with identification of balance, musculoskeletal, deconditioning, and cardiac-related reasons to exclude step testing and distinguished both cancer-related and non-cancer-related co-morbidities relevant to exercise prescription. The majority of survivors presented to clinic with appropriate indications for exercise intervention, with documentation of significant increases in sedentary activity and demonstration of average or below age- and gender-adjusted relative fitness ratings. The set-up was consistent with AHA recommendations for health assessments in the youth population, effectively incorporating evaluation of survivors of cancer into a standard visit, with theoretical benefit for all stakeholders and a format that could improve utilization of wellness initiatives promoted by insurers including Medicare^40^. Provision of fitness testing in the office enabled a hands-on experience with direct observation and demonstration. Muscle group testing in office enabled inclusion of appropriate muscle group strength building exercises into the exercise prescriptions. Survivors of cancers could themselves appreciate, in concert with the prescribing clinician, which specific muscle groups showed relative weaknesses. They were then provided the rationale for specific strengthening exercises, which were first demonstrated to them and then performed under observation for refinement of technique. In-office demonstration of other tasks, like insulin injection and inhaler use, increases patient adherence^41,42^. The format is compatible with addition of telemedicine for follow-up visits^43^.

A challenge for the study was the absence of standards for rating relative fitness utilizing step test formats appropriate to well-motivated non-geriatric survivors who need testing with a format more vigorous than a walk test^33^. Performance on these modified step tests, particularly the slower and shorter tests, was over-rated in regards to fitness level for the general population. Development of appropriate age- and gender-adjusted fitness ratings for the cancer survivor population, who frequently require shorter and/or slower tests, would facilitate cost-effective evaluation in the clinic and provide a platform for study of cardiovascular fitness in this population across settings, as the established Harvard Step Test has done for non-cancer populations. The established walk test would have been an alternative for the step test performed on the flat surface. From a practical standpoint there are advantages to a test that can be performed within the restrictions of a normal-sized examining room, but, like the elevated step tests, standards need to be established.

Optimal timing of medical clearance and exercise prescription for survivors of cancer has not been established and survivors with metastatic disease can require additional guidance based on impact and site of metastatic disease^1,3,16–19^. In this clinic, open to all survivors through either self or physician referral, the majority of survivors of cancer presented within two years of diagnosis, with 28% on chemotherapy and 48% on anti-hormonal therapy. Nine percent presented with distant metastatic disease. The format used enabled all of these survivors to engage in exercise testing and receive an exercise prescription. The significantly lower fitness ratings observed for survivors on chemotherapy that completed the 3-min 9-in. step test at pace were not unexpected but did not preclude exercise prescription^44^. Other variables correlated with fitness ratings in this population paralleled those associated with the general population. Higher BMI and initial pulse inversely correlated, and age and vigorous exercise history positively correlated with higher fitness ratings^29,45^.

Study limitations included retrospective chart review design, over-representation of female survivors of breast cancer and individuals with higher socioeconomic status as indicated by the high percentages with established residencies and employment in economic sectors associated with professional education, and conduct in a single urban National Cancer Institute-designated comprehensive cancer center with care and services for cancer patients as well as community outreach and education programs. A significant minority (29%) reported living alone with the majority (66%) describing themselves as living with family and/or life partners, possible sources of social support.

Higher socioeconomic status is significantly associated with lower rates of obesity^46^. In the U.S. obesity is defined as a BMI at or above 30.0 kg/m^2^. Obesity is a cancer risk factor that acts through different mechanisms, including inflammation^47^. Cardiovascular exercise can reduce inflammatory markers and as well as impacting other obesity-related metabolic factors associated with increased cancer risk^48,49^. Here, the overall obesity rate was 31%, comparatively lower than the 39.7% and 43.3% reported prevalence in US women aged 20–39 and 40–60 and over respectively (2017–2018)^50^, but comparable to the overall obesity prevalence of 31.7% reported in US survivors of cancer (1997–2014)^51^. We found that step test modifications to a shorter or even flat self-paced step enabled individuals of higher BMI to complete a cardiovascular fitness evaluation in office, thus enabling appropriate cardiovascular exercise prescription. We also found that a significant proportion (25%) of survivors of cancer completing the step test at pace had a BMI in the obesity range (> 30, observed range 30–44). Taken together, these results illustrate the importance of having an approach that encompasses accessibility, safety, comfort and engagement while not pre-judging ability to complete a specific step test format simply based on BMI. Having modifiable step tests is a tactic that is transferrable to clinic settings where obesity prevalence might be even higher than reported in our population.

## Methods

All methods were carried out in accordance with relevant guidelines and regulations. A retrospective chart review of all patients presenting to the Lombardi Fitness and Metabolism Clinic between 2008 and 2018 was conducted. The clinic was open to all survivors of cancer and patients attended by both self and physician referral. This study was approved by the Institutional Review Board of the Office of Research Oversight/Regulatory Affairs, Georgetown University and determined to impose minimal risk on participants. A waiver of consent was obtained from the Institutional Review Board of the Office of Research Oversight/Regulatory Affairs, Georgetown University.

### Eligibility

In order to be included in the study, patients needed to be survivors of cancer presenting for their first visit between 2008 and 2018. One hundred and sixty-nine of 171 patients (99%) met the selection criteria. Two patients seen in the clinic during this time were excluded from analysis as not having been diagnosed with cancer (n = 1 prophylactic bilateral mastectomy for elevated breast cancer risk; n = 1 chronic pulmonary disease.).

### Data collection and step test procedures

Detailed chart reviews in the Aria and Powerchart Electronic Medical Records were conducted to ascertain demographic factors (sex, age, employment status, occupation, residence, living situation), clinical characteristics (cancer type, pathologic stage, site of metastases, age at diagnosis, treatment history, BMI, resting pulse, systolic blood pressure), and fitness measures (self-reported past and current exercise histories, step test completion, pulse recovery, core testing, exercise prescription) at the time of initial office visit. Self-reported exercise histories were obtained from the time of adolescence through time of presentation at clinic. Past exercise history was defined as modes of exercise the survivor of cancer engaged in during their lifetime and was classified by intensity category as to the maximal MET level performed (sedentary, light, moderate, or vigorous) ^27,52^. Current exercise history was defined as modes of exercise the survivor of cancer was engaged in at the time of assessment and was coded by intensity category as to the maximal MET level performed (sedentary, light, moderate, or vigorous). All patients underwent a comprehensive H&P prior to office fitness evaluation. Occupations were categorized by economic sector. Cancer types were classified into five organ systems (Breast; GI including Pancreatic, Gastric, Ampulla of Vater, Rectal; Head and Neck including Tongue, Tonsil, Merkel Cell; GU including Ovary, Prostate, Renal Cell; Hematopoietic including Lymphoma, Angiosarcoma, Multiple Myeloma). Presence of distant metastasis was defined as spread from primary tumor to distant organs or distant lymph nodes. Surgical procedures related to diagnosis and/or treatment of the primary cancer were recorded. Data was stratified by the type of submaximal cardiovascular fitness assessment performed: Completed At Pace (96 ± 0 steps/min, 9 in.) (n = 88), Completed Slower Self-Paced (85 ± 1.1 steps/min, 9 in.) (n = 24), Completed Shorter Self-Paced (91 ± 1.5 steps/min, 9 in.) (n = 21), Flat Self-Paced (94 ± 1.6 steps/min) (n = 9) or whether it was contraindicated (n = 20) or deferred (n = 7). For those that completed a 9-in. step test, age and 1-min pulse recovery were used to assign YMCA Fitness Category for patients who completed step testing (Very Poor-Poor, Below Average, Average, Above Average, Good, Excellent)^29^. The validity of utilizing a step test for assessing fitness was initially established in 1943 (28) with age and gender-adjusted standards for fitness ratings published by the YMCA in 1989 (29). While the YMCA step height is 12 in., previous investigations have determined that lower heights (7.9 to 8 in.) are easier to perform for individuals with shorter leg lengths, older age, and/or physical impairment, can provide a validated fitness assessment, and are more reflective of the step height range mandated by building codes (reviewed in ref 32). Here we used a 9-in. step because the height is standard for medical step stools approved for use in clinical environments and lies within the range of heights used in previously published studies (7.9–12 in.). In order to perform the test, individuals were initially cleared to be able to attempt the step test by H&P exam. If the H&P revealed active pain (n = 11), severe deconditioning or chemotherapy related severe fatigue (n = 4), indications for cardiac stress test (n = 2), onycholysis (n = 1), wound dehiscence n = 1), or balance impairment (n = 1), a step test was deemed contraindicated. Balance was specifically assessed in the physical exam through tests of coordination, gait and/or use of the Rhomberg Test because safe performance of the step test required adequate balance. When H&P and patient records revealed adequate information to assess cardiovascular fitness (n = 6) or the patient needed to leave the appointment early to attend another medical appointment (n = 1), the step test was deemed deferred. For performance of the step test, first the rationale, equipment, and procedure including the fact that the test will not exceed 3 min, was introduced to the individual through a verbal explanation followed by a demonstration of how to step up and down on the step at a pace of 96 steps/min with instructions that they should not speak during the procedure, that they will be informed of time elapsed at 30 s increments and, to promote safety, comfort and engagement, that they may either slow down their pace (e.g. self-pace) or stop at any time they desired either because of a specific (for example pain, breathlessness, fatigue) or general (feeling of discomfort) reason. Following the stepping they were informed that they should sit in the chair provided and that their pulse would be recorded by either radial pulse or heart auscultation for one full minute following the test, and that the results and their relative fitness rating based on the step test would be reported to them immediately. The pace that each individual actually performed the test was recorded. N = 88 (52%) of the individuals attempting the 9-in. step test were able to complete 3 min at pace (96 steps/min). N = 24 (14%) were able to complete 3 min but did so at a slower self-pace. N = 21 (12%) stopped the test before 3 min with n = 13 completing at pace and n = 8 completing at a slower self-pace. During the H&P nine individuals were identified as having contraindications to performing a 9-in. step test but adequate physical capability to perform stepping on a flat surface (n = 7 chronic knee pain; n = 2 impaired balance). Results of core strength testing (numbers of crunches and/or modified push-ups from the knees, squat testing (normal defined as symmetric performance of five serial squats) and plank duration were recorded for each category of cardiovascular fitness assessment including those for which a step test was contraindicated or deferred^34–36^.

### Statistical analyses

Mean, standard deviation, and range were calculated for years since diagnosis, age at clinic presentation, age at diagnosis, BMI, initial office pulse, and initial office systolic blood pressure (GraphPad Prism version 9.0.0 for macOS, GraphPad Software, San Diego, California USA, http://www.graphpad.com). Associations between categorical variables were analyzed using Chi Square (https://www.socscistatistics.com/tests/chisquare2/default2.aspx) and Fisher’s Exact (http://vassarstats.net/fisher2x4.html; https://astatsa.com/FisherTest/) tests (accessed July–September 2020). One-way ANOVA followed by Tukey’s multiple comparisons test was performed to examine associations between continuous and categorical variables (GraphPad Prism). Associations between two continuous variables were analyzed using linear regression (GraphPad Prism 9.0.0). For all tests, *p* values equal to or less than 0.05 were considered statistically significant with actual* p* values derived reported.

### Research involving human participants

This study was approved by the Institutional Review Board (IRB) of the Office of Research Oversight/Regulatory Affairs, Georgetown University. It was determined to impose minimal risk on participants. A waiver of consent was obtained.

### Informed consent

A waiver of consent was obtained.

### Ethical approval

This study was approved by the Institutional Review Board (IRB) of the Office of Research Oversight/Regulatory Affairs, Georgetown University.
